# Methamphetamine-mediated endoplasmic reticulum (ER) stress induces type-1 programmed cell death in astrocytes via ATF6, IRE1α and PERK pathways

**DOI:** 10.18632/oncotarget.10025

**Published:** 2016-06-14

**Authors:** Ankit Shah, Anil Kumar

**Affiliations:** ^1^ Division of Pharmacology and Toxicology, School of Pharmacy, University of Missouri-Kansas City, Kansas City, MO 64108, USA

**Keywords:** methamphetamine, astrocytes, ER stress, CHOP, apoptosis

## Abstract

Methamphetamine (MA), a psychostimulant drug has been associated with a variety of neurotoxic effects which are thought to be mediated by induction of pro-inflammatory cytokines/chemokines, oxidative stress and damage to blood-brain-barrier. Conversely, the ER stress-mediated apoptosis has been implicated in several neurodegenerative diseases. However, its involvement in MA-mediated neurodegenerative effects remains largely unexplored. The present study was undertaken to assess the effect of MA on ER stress and its possible involvement in apoptosis. For this purpose, SVGA astrocytes were treated with MA, which induced the expressions of BiP and CHOP at both, mRNA and protein levels. This phenomenon was also confirmed in HFA and various regions of mouse brain. Assessment of IRE1α, ATF6 and PERK pathways further elucidated the mechanistic details underlying MA-mediated ER stress. Knockdown of various intermediate molecules in ER stress pathways using siRNA demonstrated reduction in MA-mediated CHOP. Finally, MA-mediated apoptosis was demonstrated via MTT assay and TUNEL staining. The involvement of ER stress in the apoptosis was demonstrated with the help of MTT and TUNEL assays in the presence of siRNA against various ER stress proteins. The apoptosis also involved activation of caspase-3 and caspase-9, which was reversed by knockdown with various siRNAs. Altogether, this is the first report demonstrating mechanistic details responsible for MA-mediated ER stress and its role in apoptosis. This study provides a novel group of targets that can be explored in future for management of MA-mediated cell death and MA-associated neurodegenerative disorders.

## INTRODUCTION

Methamphetamine (MA) is a potent psychostimulant drug that is widely abused worldwide including United States. A 2013 study by National Survey on Drug Use and Health (NSDUH) has shown that over 12 million people (4.7 percent of the population) have tried methamphetamine at least once [1]. Its effect usually lasts for 10-12 hours and it causes an increase in the release of epinephrine, serotonin, and a decrease in dopamine as well as dopamine transporter (DT) [2–7]. MA also causes alterations in brain structure including gray matter loss, neuronal damage and microgliosis in various regions of the brain [8–10]. These changes in the brain have been attributed to oxidative stress [11], alteration in blood-brain barrier (BBB) integrity [12], mitochondrial dysfunction [13] and increased excitotoxicity [14]. Recently, the data from our laboratory have also reported increase in proinflammatory cytokines and involvement of cytochrome P450 in MA-mediated oxidative stress as other possible mechanisms for MA-mediated neurotoxicity. [15, 16].

Among various neurotoxic outcomes, MA abuse also induces apoptosis in a variety of brain cells [16–18]. Apoptosis is a highly regulated process to induce cell death, which involves complex signaling cascades and stress responses [19]. Several studies have demonstrated that increased oxidative stress, DNA damage, endoplasmic reticulum (ER) stress, heat shock proteins and mitochondrial stress serve as underlying mechanisms to induce apoptotic cell death [20].

ER stress is a transient process that enables cells to overcome the abnormal accumulation of unfolded/misfolded proteins [21, 22]. It has been implicated in several diseases such as Parkinson's disease, ischemia, atherosclerosis and diabetes [23–25]. In addition, increased ER stress has been correlated with MA-mediated neurotoxicity [13, 26–28]. Several reports have demonstrated increased expressions of various ER stress markers and chaperones due to MA leading to neuronal death. However, the underlying mechanism responsible for MA-mediated ER stress is not fully elucidated yet. The ER stress is a complex process that involves activation of 3 major signaling pathways: Activating transcription factor 6 (ATF6), Inositol-requiring enzyme-1α (IRE1α) and Protein kinase RNA-like endoplasmic reticulum kinase (PERK). In response to the accumulation of unfolded proteins in the ER, BiP is released from IRE1α, PERK and ATF6 to chaperone the accumulated proteins to degradation via ubiquitination [22]. The BiP-free ER stress sensors are differentially activated to initiate their downstream cascades leading to increase in C/EBP homologous protein (CHOP), indicating increased ER stress. Moreover, increased expression of CHOP has been reported to activate apoptosis in various studies [23, 29–31].

Among various cells in the brain, astrocytes perform a variety of maintenance and regulatory functions such as regulation of glutamatergic signaling, maintenance of metabolites and extracellular ions, synaptic maintenance and structural support [32]. Since astrocytes contribute to about 40-60% of the cells in the brain, it is important to understand the indirect neurotoxicity resulting from astrocytes. Studies have demonstrated increased ER stress in astrocytes via PERK activation, which further leads to increase in oxidative stress and inflammation via various signaling pathways [33, 34]. The increased ER stress in astrocytes has been implicated in Alzheimer's disease [35] and Parkinson's disease [36]. Thus, it is important to understand the effect of MA on astrocytic ER stress.

In the present study, we sought to address the underlying mechanism(s) responsible for MA-mediated ER stress. We further extended our study to understand the effect of MA-mediated ER stress on apoptosis.

## RESULTS

### MA induced the expressions of ER stress markers in astrocyte cell-line in time and dose–dependent manner

**Figure 1:**
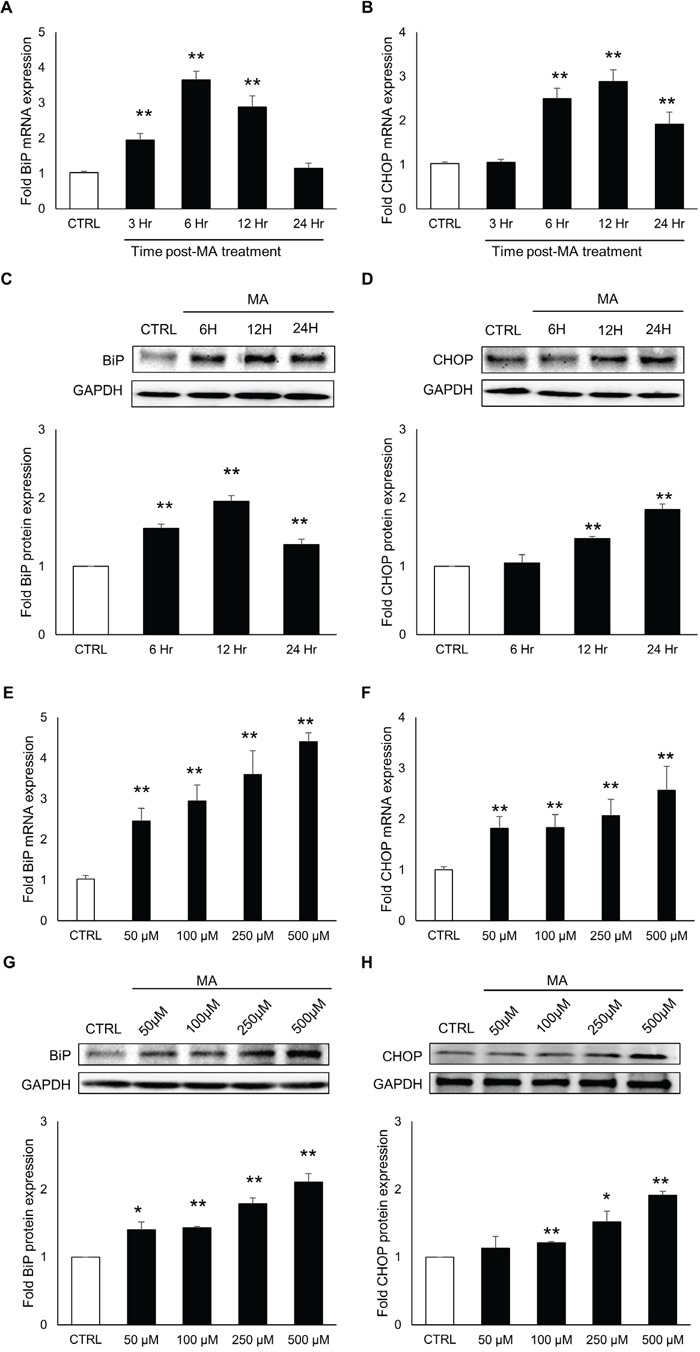
MA induced the expressions of ER stress markers in time and concentration-dependent manner **A-H.** SVGA cells were seeded in 12-well plates at 2.5 × 10^5^ cells/well in complete DMEM medium. The cells were incubated overnight for adherence and treatments with MA were performed. (A-D) Cells were treated with 500 μM MA for 3, 6, 12 and 24 H and RNA was isolated using QIAGEN RNeasy kit. The levels of BiP (A) and CHOP (B) were measured using real time RT-PCR and the values represented in the bar graphs were calculated with respect to untreated control. Similarly, whole cell lysates were prepared and the protein levels of BiP (C) and CHOP (D) were measured at 6, 12 and 24 hours. The fold protein expressions were calculated with respect to untreated control. (E-H) Cells were treated with 50, 100, 250 and 500 μM MA and the RNA levels of BiP (E) and CHOP (F) were measured at 6 and 12 H, respectively. Similarly, protein levels of BiP (G) and CHOP (H) were measured at 12 and 24 H, respectively. Fold RNA and protein expressions were calculated with respect to untreated controls. The RNA and protein expressions in all the experiments were normalized with *HPRT* and *GAPDH* as housekeeping genes, respectively. The results shown in bar graphs were obtained from at least 3 independent experiments with each treatment performed in triplicates. The bar graphs shown in the figure are represented in mean ± S.E., while the western blots are representative images. Statistical significance was calculated using one-way ANOVA with multiple comparisons and the values were considered significant if p-value ≤ 0.05 (*) or ≤ 0.01 (**).

As a response to the ER stress, cells produce increased levels of BiP and CHOP, which are key markers of ongoing ER stress [37]. The continuous accumulation of these proteins lead to apoptosis. Therefore, we decided to measure the levels of BiP and CHOP over a period of 24 hours after MA treatment. For this purpose, SVG astrocytes were treated with 500 μM MA for 3, 6 12 and 24 hours. Total RNA and whole cell lysates were prepared and the mRNA and protein levels of BiP and CHOP were quantified using real-time RT-PCR and western blotting, respectively. As shown in Figure 1A-1B, the mRNA levels of BiP and CHOP increased over time and the peak levels were observed at 6H for BiP (3.7 ± 0.2 fold) and at 12H for CHOP (2.9 ± 0.2 fold). Similarly, the protein levels of BiP and CHOP were measured using western blotting (Figure 1C-1D) and the peak levels of BiP (2.0 ± 0.1 fold) and CHOP (1.8 ± 0.1 fold) were observed at 12H and 24H, respectively. Having determined the optimum time for the maximum response, we determined the increase in the levels of BiP and CHOP at various concentrations of MA. For the purpose, SVGA cells were treated with 50, 100, 250 and 500 μM of MA. The mRNA levels of BiP and CHOP were measure at 6 H and 12 H post-treatment, respectively (Figure 1E-1F). The peak level of BiP and CHOP RNA were observed in cells treated with 500 μM MA (4.4 ± 0.2 fold for BiP and 2.6 ± 0.5 fold for CHOP). Similarly, the protein levels of BiP and CHOP were assessed at 12 H and 24 H, respectively, which showed peak expressions (2.1 ± 0.1 and 1.9 ± 0.1 fold for BiP and CHOP, respectively) with 500 μM MA (Figure 1G-1H). Together, these results indicated a dose and time-dependent increase in the expressions of BiP and CHOP at both, mRNA and protein levels. Therefore, all the following studies in SVGA cell-line were conducted with these optimal time points (6 H for RNA and 12 H for protein). To test the effect of single dose of MA on ER stress, we chose 500 μM MA for rest of our experiments. This dose of MA was based on the blood concentrations and tissue/serum compartmentalization as reported in literatures [38–40]. Furthermore, the binge administration of MA in the range of 250 mg–1 g has been found to produce brain concentrations of MA between 164 and 776 μM [39].

### MA-mediated ER stress involved activation of ATF6 pathway

**Figure 2:**
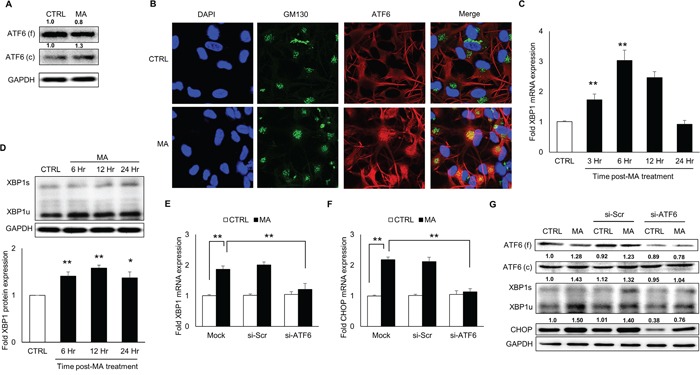
MA-mediated ER stress involved activation of ATF6 pathway SVGA cells were seeded in 12-well plates at 2.5 × 10^5^ cells/well and treated with 500 μM MA for 12 H unless specified otherwise. For all the experiments involving siRNA transfection, the cells were seeded in 6-well plate and transfected with various siRNA as described in materials and methods prior to treatment with MA. **A.** The SVGA cells were treated with MA for 12 H and whole cell lysates were prepared. The levels of ATF6 were detected using western blotting using appropriate antibody to detect the cleaved and full form of ATF6. **B.** Astrocytes were grown on glass coverslips and immunocytochemistry was performed at 6 H after treatment with MA as described in materials and methods. Briefly, the cells were fixed with methanol and stained with appropriate antibodies for ATF6 (red), GM130 (green) and the nuclei were counterstained with DAPI (blue). The cells were also stained with GFAP as astrocyte marker (data not shown). The images were captured using confocal microscope (Leica TCS SP5 II) at 40X zoom. Different channels were merged to obtain co-localization of ATF6 with golgi marker - GM130, to demonstrate activation of ATF6. **C.** Cells were treated with MA for 3, 6, 12 and 24 H and RNA were isolated. The RNA expressions of XBP1 were measured using real time RT-PCR and fold expressions were calculated with respect to untreated control. **D.** Similarly, whole cell lysates were prepared at 6, 12 and 24 H and protein levels of XBP1 were measured. **E-G.** The ATF6 was knocked down using siRNA (as mentioned in materials and methods) and the effect on XBP1 (E) and CHOP (F) RNA were measured using real time RT-PCR. Similarly, the effect of ATF6 knock down was determined on cleaved ATF6, XBP1 and CHOP proteins using western blotting (G). The RNA and protein expressions in all the experiments were normalized with *HPRT* and *GAPDH* as housekeeping genes, respectively. The results shown in bar graphs were obtained from at least 3 independent experiments with each treatment performed in triplicates. The bar graphs shown in the figure are represented in mean ± S.E., while the western blots are representative images. The numbers above the blots represent mean intensity of the respective bands. Statistical significance was calculated using one-way ANOVA with multiple comparisons and the values were considered significant if p-value ≤ 0.05 (*) or ≤ 0.01 (**).

In order to determine whether ATF6 activation was involved in MA-mediated ER stress, we measured the levels of ATF6 in the MA-treated SVGA astrocytes. As shown in Figure 2A, MA increased the cleaved ATF6 while marginally reducing the full ATF6. Since the activation of ATF6 involves translocation of ATF6 from the ER lumen to golgi, we determined whether MA treatment would result in ATF6 accumulation in the golgi. In order to do this, cells were treated with MA for 6 hours and ATF6 was stained for its organelle compartmentalization using GM130 as golgi marker. As shown in Figure 2B, treatment with MA resulted in increased accumulation of ATF6 in golgi as observed by co-localization of ATF6 with GM130. Once transported into golgi, ATF6 is proteolytically cleaved to release the cytosolic ATF6, which then shuttles into the nucleus to serve as a transcription factor [41] to increase the levels of X-box binding protein 1 (XBP1) and CHOP. To further confirm the activation of ATF6 in MA-mediated ER stress, we determined the expressions of XBP1 at mRNA and protein levels (Figure 2C-2D). MA treatment increased the mRNA and protein levels of XBP1 by 3.0 ± 0.3 fold (Figure 2C) and 1.6 ± 0.1 fold (Figure 2D), respectively. Finally, we used siRNA to knockdown the expressions of ATF6, which resulted in reduction of both, XBP1 and CHOP at mRNA (Figure 2E-2F) and protein (Figure 2G) levels. Altogether, these results demonstrated that MA-mediated ER stress involved activation of ATF6, which led to increase in the expressions of CHOP and XBP1.

### MA-mediated ER stress involved activation of IRE1α pathway

**Figure 3:**
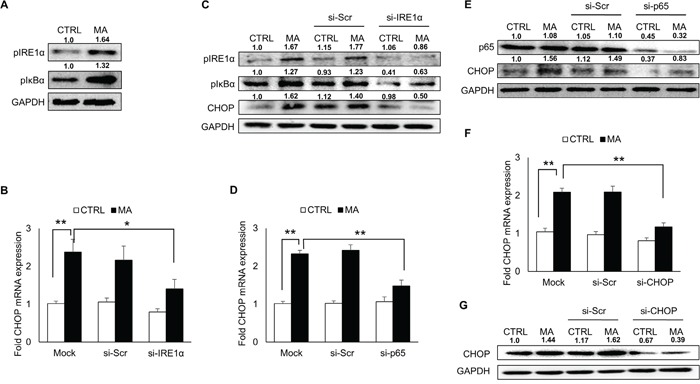
Involvement of IRE1α pathway in MA-mediated ER stress SVGA cells were seeded in 12-well plates at 2.5 × 10^5^ cells/well and treated with 500 μM MA for 12 H. For all the experiments involving siRNA transfection, the cells were seeded in 6-well plate and transfected with various siRNA as described in materials and methods prior to treatment with MA. **A.** Treatment with MA resulted in increased levels of phosphorylated IRE1α and phosphorylated IκBα indicating their activation. **B-G.** To confirm their involvement in MA-mediated ER stress, various siRNAs were employed to knockdown their target genes as mentioned in materials and methods. (B-C) The effect of IRE1α knockdown using specific siRNA was determined on MA-mediated CHOP mRNA (B) and protein (C) expressions. (C) Knockdown of IRE1α also reduced the expression of phosphorylated IRE1α and phosphorylated IκBα. (D-E) To confirm the activation of NF-κB, p65 was knocked down using siRNA and the effect on CHOP mRNA (D) and protein (E) expressions. (F-G) The effect of CHOP knockdown was assessed on CHOP mRNA (F) and protein (G) levels. The RNA and protein expressions in all the experiments were normalized with *HPRT* and *GAPDH* as housekeeping genes, respectively. The results shown in bar graphs were obtained from at least 3 independent experiments with each treatment performed in triplicates. The bar graphs shown in the figure are represented in mean ± S.E., while the western blots are representative images. The numbers above the blots represent mean intensity of the respective bands. Statistical significance was calculated using one-way ANOVA with multiple comparisons and the values were considered significant if p-value ≤ 0.05 (*) or ≤ 0.01 (**).

Under stressed environment, IRE1α situated in the ER transmembrane is activated via oligomerization followed by its phosphorylation [22]. To assess whether MA-mediated ER stress involved activation of IRE1α pathway, we measured the levels of phosphorylated IRE1α (Figure 3A), which was elevated upon treatment of SVGA cells with MA. In order to confirm the involvement of IRE1α in MA-mediated ER stress, we measured the expressions of CHOP after silencing IRE1α. The knockdown of IRE1α resulted in reduction of MA-mediated CHOP expressions at RNA (Figure 3B) and protein (Figure 3C) levels. The IRE1α activation also involves several downstream mechanisms leading to production of CHOP, which include activation of c-Jun N-terminal kinase (JNK)/activator protein 1 (AP-1), activation of nuclear factor kappa B (NF-κB) and splicing of XBP1 [22, 42, 43]. We found that MA treatment neither resulted in activation of JNK via phosphorylation (data not shown) nor increase in spliced XBP1 (Supplementary Figure S2). Therefore we investigated whether NF-κB activation was involved in downstream signaling of IRE1α activation. Our results indicated that phosphorylated IκBα levels were increased in MA-treated astrocytes (Figure 3A) suggesting NF-κB activation. In addition, when we knocked down the expressions of IRE1α, we observed reduction in the levels of pIκBα, which confirmed that phosphorylation of IκBα resulted from the activation of IRE1α (Figure 3C). To further confirm these findings, we silenced p65 with specific siRNA, which resulted in reduction of CHOP at mRNA and protein levels (Figure 3D-3E). Finally, the expressions of CHOP at mRNA (Figure 3F) and protein (Figure 3G) levels were reduced with siRNA against CHOP. These results clearly indicated the involvement of IRE1α pathway in MA-mediated ER stress, which also involved activation of NF-κB, which then increased CHOP.

### MA-mediated ER stress involved activation of PERK pathway

**Figure 4:**
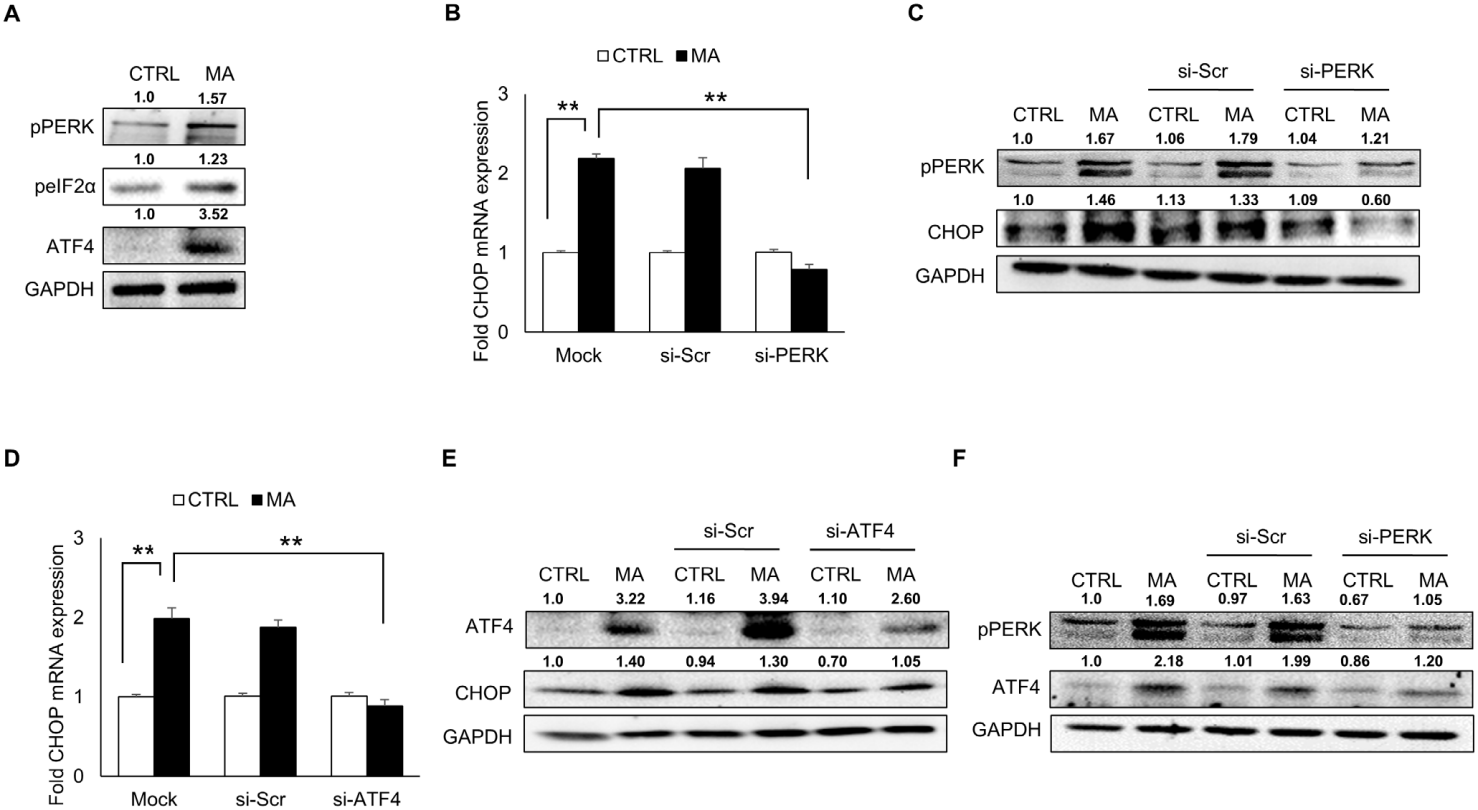
MA-mediated ER stress involved activation of PERK pathway SVGA cells were seeded in 12-well plates at 2.5 × 10^5^ cells/well and treated with 500 μM MA. For all the experiments involving siRNA transfection, the cells were seeded in 6-well plate and transfected with various siRNA as described in materials and methods prior to treatment with MA. **A.** The levels of phosphorylated PERK, phosphorylated eIF2α and ATF4 were increased as a result of MA treatment. **B-C.** Knockdown of PERK using siRNA reduced the levels of MA-mediated CHOP mRNA (B) and protein (C). **D-E.** Similarly, knockdown of ATF4 also reduced the CHOP mRNA (D) and protein (E). **F.** The levels of ATF4 were measured after knockdown of PERK to demonstrate a link between PERK activation and increase in ATF4 (F). The RNA and protein expressions in all the experiments were normalized with *HPRT* and *GAPDH* as housekeeping genes, respectively. The results shown in bar graphs were obtained from at least 3 independent experiments with each treatment performed in triplicates. The bar graphs shown in the figure are represented in mean ± S.E., while the western blots are representative images. The numbers above the blots represent mean intensity of the respective bands. Statistical significance was calculated using one-way ANOVA with multiple comparisons and the values were considered significant if p-value ≤ 0.01 (**).

Having demonstrated the involvement of ATF6 and IRE1α pathway in MA-mediated ER stress, we sought to address whether PERK activation could also serve as a mechanism for MA-mediated ER stress. In order to demonstrate this, SVGA cells were treated with MA and the expressions of phosphorylated PERK were measured. As shown in Figure 4A, MA treatment increased the expressions of phospho PERK, and phospho eIF2α. In addition, earlier reports have demonstrated increase in ATF4 in rat striatum due to MA treatment [28]. Since ATF4 activation is downstream of PERK pathway, we measured the levels of ATF4. As shown in Figure 4A MA increased the expression of ATF4, which was reduced by knocking down PERK using siRNA (Figure 4F). Moreover, silencing of PERK and ATF4 also reduced the expressions of CHOP at mRNA (Figure 4B and 4D, respectively) and protein (Figure 4C and 4E, respectively) levels. Altogether, these results suggested that MA activated PERK, which led to increase in ATF4 and thereby increased expressions of CHOP.

### MA induced the expressions of ER stress markers in primary human fetal astrocytes

**Figure 5:**
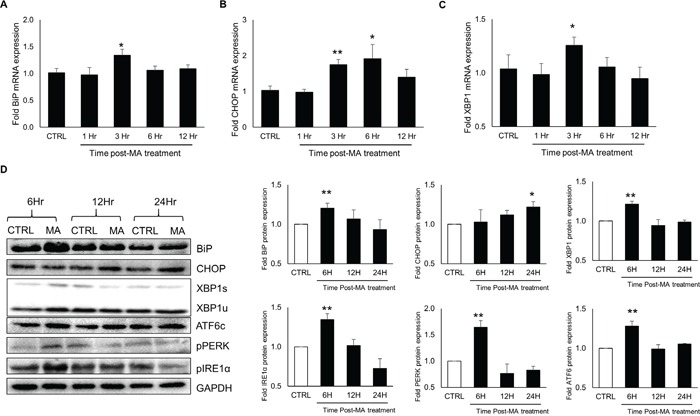
MA induced the expressions of ER stress markers in primary human fetal astrocytes HFA were seeded at 1 × 10^6^ cells/well in 12-well plates and treated with 500 μM MA for 1, 3, 6 and 12 hours. The RNA expressions of BiP **A.**, CHOP **B.** and XBP1 **C.** were measured using real time RT-PCR. Similarly, the protein levels **D.** of BiP, CHOP and XBP1 were measured after 6, 12 and 24 hours. The levels of IRE1α, ATF6 and PERK were determined in the protein lysates (D). The expression values were normalized with GAPDH as loading control and fold expressions were calculated with respect to untreated control for respective time. The results were obtained from at least 3 different donors with each treatment performed in triplicates and the bar graphs shown in the figure are represented in mean ± S.E. The western blots are representative images, which were obtained by cutting the membranes between the molecular weights of protein of interest using molecular markers. Statistical significance was calculated using one-way ANOVA with multiple comparisons and the values were considered significant if p-value ≤ 0.01 (**) or ≤ 0.05 (*).

To verify results obtained in SVGA cell-line, we assessed the effect of MA treatment in HFA. Briefly, HFA were treated with 500 μM MA for 1, 3, 6 and 12 hours for RNA and 6, 12 and 24 hours for protein expressions of various ER stress markers. The RT-PCR performed for RNA levels demonstrated (Figure 5A-5C) marginal but significant increase in BiP (1.3 ± 0.1 fold) and XBP1 (1.3 ± 0.1 fold) at 3 H, while considerable increase in CHOP at 3-6 H with peak expressions at 6H (1.9 ± 0.4 fold) after MA treatment. Similarly, the protein levels (Figure 5D) of BiP and XBP1 increased after MA-treatment for 6 H (1.21 ± 0.06 fold for BiP and 1.22 ± 0.04 fold for XBP1) and CHOP levels increased at 24 H (1.22 ± 0.07 fold). Moreover, we also tested whether the increased levels of these molecules involved the major ER-stress initiator proteins. As shown in Figure 5D, MA treatment resulted in increased levels of pIRE1α (1.35 ± 0.07 fold), pPERK (1.64 ± 0.13 fold) and ATF6c (1.28 ± 0.06 fold) at 6H, suggesting involvement of all three activator proteins.

### Acute treatment of MA induced the expressions of ER stress markers in various brain regions of mice

**Figure 6:**
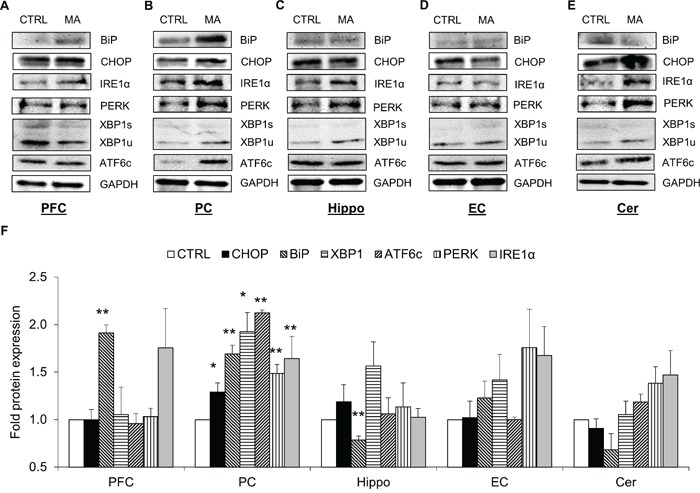
MA induced the expressions of ER stress markers in various brain regions of mice Male C57BL/6 mice at the age of 14-16 weeks (n=4) were given 4 s.c. injections with each separated 2 hours apart of either saline or 10mg/kg MA. The brains of the mice were isolated after 24 hours from the last injection and subdivided into PFC **A.**, PC **B.**, Hippocampus **C.**, EC **D.**, and Cerebellum **E.** to prepare protein lysates. The proteins from each regions from the respective treatment groups were pooled in equal amounts and the western blots were performed to determine the levels of BiP, CHOP, XBP1, ATF6, IRE1α, and PERK. The protein expressions in all the experiments were normalized with *GAPDH* as housekeeping gene. The results shown in bar graphs **F.** were quantified from at least 3 independent experiments and expressed as mean ± S.E. The blots are representative images. Statistical significance was calculated using one-way ANOVA with multiple comparisons and the values were considered significant if p-value ≤ 0.01 (**) or ≤ 0.05 (*).

To verify the results obtained with our *in vitro* experiments, we determined the levels of various ER stress proteins in the brains of C57BL/6 mice after MA treatment. The mice were treated with four s.c injections of 10 mg/kg MA with each injection 2 hour apart. This dose of MA was chosen based on previously published reports, where 10-40 mg/kg of MA was found to be neurotoxic via various mechanisms [13, 44, 45]. The brains of mice were collected after 24 hours from the last MA injection and protein lysates were prepared from PFC, PC, hippocampus, EC, and cerebellum (Figure 6A-6E). Assessment of protein levels in different regions demonstrated alteration of various ER stress proteins at variable extents. Among various regions, PC showed most consistent results with respect to all the ER stress proteins. In addition to PC, PFC and Hippocampus showed significant changes in the BiP levels (Figure 6). The other regions of the brain demonstrated moderate changes in the ER stress protein, which were statistically insignificant (Figure 6F). Overall, these results confirmed the results obtained with the *in vitro* experiments suggesting ER stress induction due to MA.

### MA-mediated ER stress increased cell death in astrocytes

**Figure 7:**
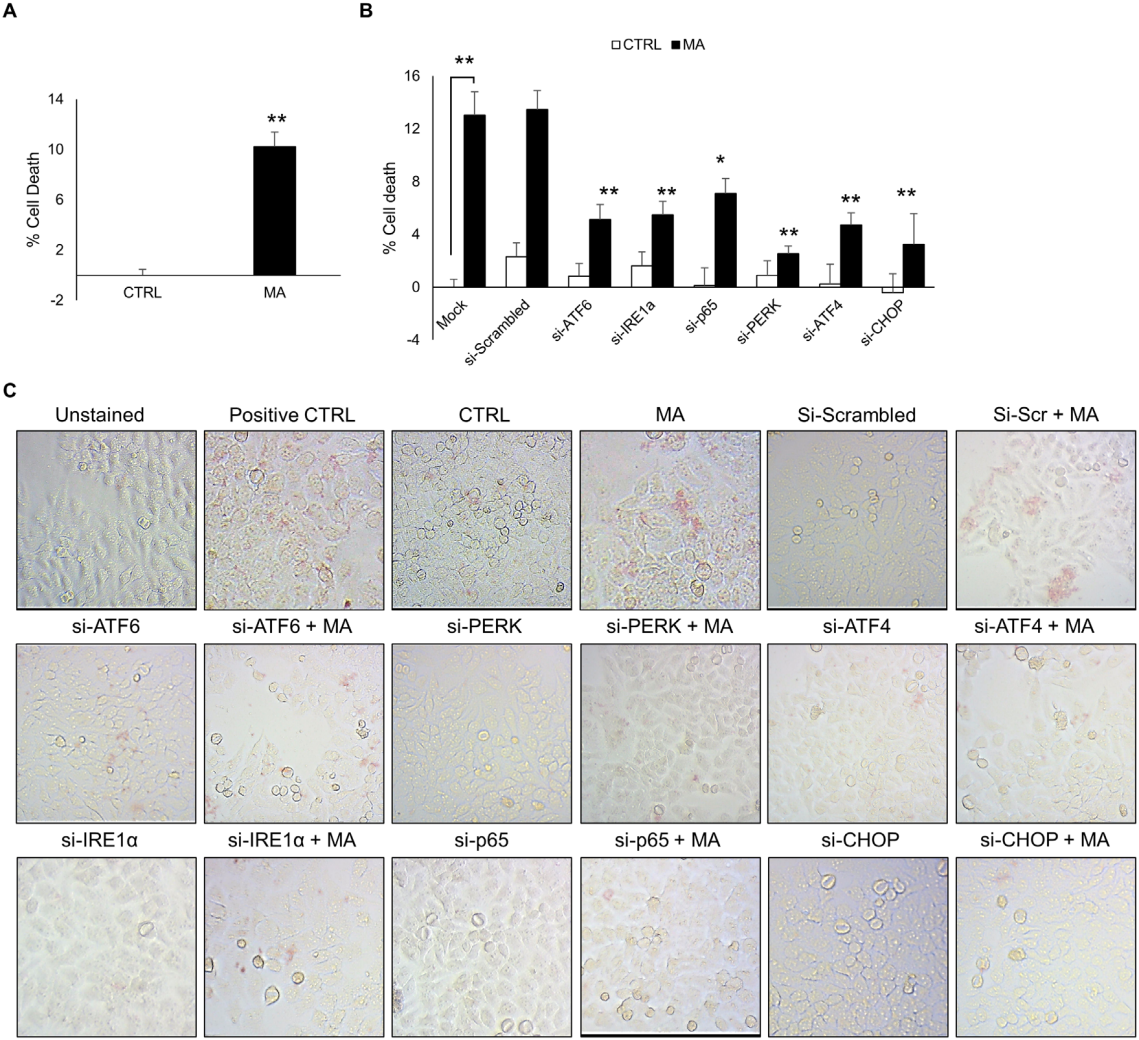
MA-mediated ER stress increased cell death in astrocytes Cell death was measured at 48 hours after the treatment with 500 μM MA. Briefly, SVGA cells were seeded at 6 × 10^5^ cells in a 6-well plate and transfected with various siRNA as mentioned in methods. After 48 hours, the cells were reseeded in 12-well plate and allowed to adhere overnight. Finally, these cells were treated with 500 μM MA for additional 48 H and MTT assay was performed to assess the effect of ATF6, PERK, ATF4, IRE1α, p65 and CHOP knockdown. **A.** The % cell death was calculated considering the absorbance in untreated control as 100% viability. **B.** The involvement of ATF6, IRE1α and PERK pathways and their respective intermediates leading to CHOP in cell death was assessed using MTT assay after knockdown of each of the intermediates with siRNA, which demonstrated reduction in MA-mediated cell death at variable extents. **C.** Similarly, to confirm these results, TUNEL staining was performed at 48 hours after MA treatment. The TUNEL staining was performed using TACS-XL *In Situ* Apoptosis Detection Kit - DAB kit as described in materials and methods. The images were captured at 20X zoom using Labomed iVu5100 camera. The results shown in bar graphs were obtained from at least 3 independent experiments with each treatment performed in triplicates and presented in mean ± S.E. Statistical significance was calculated using one-way ANOVA with multiple comparisons and the values were considered significant if p-value ≤ 0.05 (*) or ≤ 0.01 (**).

In order to determine the functional implication of MA-mediated ER stress, we assessed the effect of siRNAs against various molecules in ATF6, IRE1α and PERK pathway. To do so, we first measured the effect of MA on cell death using MTT assay. MA increased the cell death in astrocytes by 10.2 ± 1.2 % (Figure 7A). Having demonstrated the effect of MA on cell survival, we sought to determine whether ER stress was involved in MA-mediated cell death. Therefore, we silenced ATF6, IRE1α, p65, PERK, ATF4 or CHOP in astrocytes using siRNA. These cells were then treated with MA to assess the effect on cell death. As shown in Figure 7B, all the siRNAs reduced MA-mediated cell death at variable extents. This results were further confirmed with TUNEL staining, which demonstrated reduction in the TUNEL positive cells after MA treatment, when various ER stress molecules were silenced (Figure 7C). Altogether these results demonstrate that MA-mediated ER stress resulted in cell death in astrocytes, which was at least in part attributed to IRE1α, PERK and ATF6 pathway. Moreover, inhibition of either of these pathways could partially circumvent MA-mediated cell death.

### Role of opioid receptor in MA-mediated ER stress and cell death

**Figure 8:**
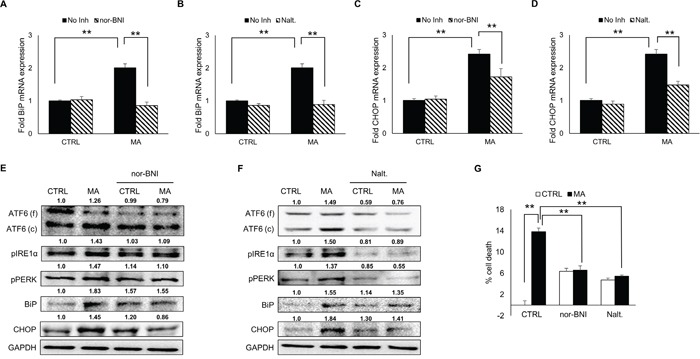
Role of opioid receptor in MA-mediated ER stress SVGA cells were pre-treated with either 20 μM of nor-BNI, a selective inhibitor for κ-opioid receptor or 10 μM of naltrexone, a general inhibitor for opioid receptor 1 H before the treatment with MA and the effect was observed on the mRNA and protein expressions of BiP and CHOP. **A, C.** Pre-treatment with nor-BNI reduced the levels of BiP (A) and CHOP (C) mRNA. **B, D.** Pre-treatment with naltrexone reduced the levels of BiP (B) and CHOP (D) mRNA. **E-F.** The involvement of opioid receptor in MA-mediated ER stress was assessed by western blotting on PERK, IRE1α, ATF6, BiP and CHOP. Both, nor-BNI (E) and naltrexone (F) reduced the levels of all these markers at variable extents. **G.** The effect of nor-BNI and naltrexone on MA-mediated cell death was assessed by MTT assay at 48 H after MA treatment. The RNA and protein expressions in all the experiments were normalized with *HPRT* and *GAPDH* as housekeeping genes, respectively. The results shown in bar graphs were obtained from at least 3 independent experiments with each treatment performed in triplicates. The bar graphs shown in the figure are represented in mean ± S.E., while the western blots are representative images. The numbers above the blots represent mean intensity of the respective bands. Statistical significance was calculated using one-way ANOVA with multiple comparisons and the values were considered significant if p-value ≤ 0.01 (**).

There is a huge body of literature demonstrating involvement of opioid receptor in MA-mediated dependence [46, 47]. In addition, κ-opioid receptor (KOR) was found be responsible for MA-mediated autophagy [48]. To assess whether opioid receptor contribute to MA-mediated ER stress, we employed naltrexone (a general opioid receptor inhibitor) and nor-BNI (KOR inhibitor). Astrocytes were pretreated with either 20 μM of nor-BNI or 10 μM of naltrexone for 1 hour prior to MA treatment. The mRNA levels of BiP (Figure 8A-8B) and CHOP (Figure 8C-8D) were quantified using real-time RT-PCR, which showed significant reduction for BiP (2.01 ± 0.12 vs 0.86 ± 0.11 fold for nor-BNI and 0.89 ± 0.12 fold for naltrexone) and CHOP (2.42 ± 0.14 vs 1.72 ± 0.25 fold for nor-BNI and 1.47 ± 0.12 fold for naltrexone). Moreover, both, nor-BNI (Figure 8E) and naltrexone (Figure 8F) reduced protein levels of cleaved ATF6, pIRE1α, pPERK and CHOP. These results indicates towards a possible interaction between opioid receptor and MA, which may contribute to ER stress. Since both, KOR inhibitor and general opioid inhibitor reduced MA-mediated ER stress, we sought to address whether use of these inhibitors could prevent MA-mediated cell death in astrocytes. Indeed, when assessed with MTT assay (Figure 8G), both nor-BNI (13.8 ± 0.7 vs 6.6 ± 0.8 %) and naltrexone (13.8 ± 0.7 vs 5.4 ± 0.3 %) significantly reduced MA-mediated cell death. Based on our findings in the present study, it is plausible to consider KOR as an important receptor for MA in astrocytes.

### Role of caspase-3 and caspase-9 in MA-mediated cell death

**Figure 9:**
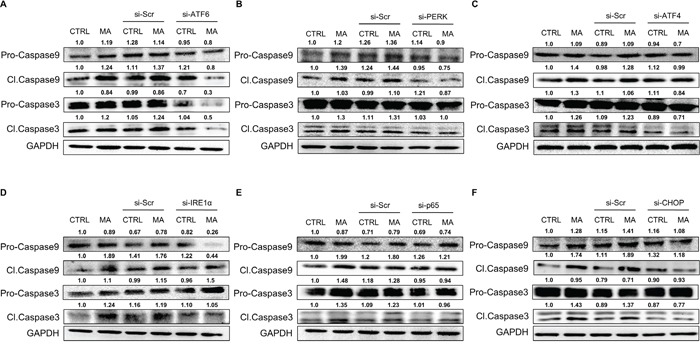
MA-mediated ER stress involved caspase-3 and caspase-9 in astrocyte cell death **A-F.** SVGA cells were seeded at 6 × 10^5^ cells in a 6-well plate and transfected with various siRNA as mentioned in methods. After 48 hours, the cells were reseeded in 12-well plate and allowed to adhere overnight. Finally, these cells were treated with 500 μM MA for additional 24 hours. The effect of specific siRNA against ATF6 (A), PERK (B), ATF4 (C), IRE1α (D), p65 (E) and CHOP (F) were assessed on the levels of cleaved and pro-caspase-3 and caspase-9 using western blotting. The western blots are representative images of at least 3 independent experiments. The numbers above the blots represent mean intensity of the respective bands.

Having demonstrated the involvement of MA-associated ER stress in cell death, we wished to determine the underlying mechanism for the same. Since caspase cascade is classically associated with apoptotic cell death in variety of cells [49] and based on our previous finding that MA-mediated cell death in astrocytes involved caspase-3 [16], we assessed the involvement of various caspases. Particularly, caspase-3 and caspase-9 were increased as a result of MA treatment (Figure 9). Since the cleaved forms of these caspases are known to be more important in the event of apoptosis, we focused on the cleaved caspase-3 & -9. As shown in Figure 9, the expressions of both, pro- and cleaved forms of caspase-3 and -9 were increased due to MA treatment. Moreover, silencing of ATF6 (Figure 9A), PERK (Figure 9B), ATF4 (Figure 9C), IRE1α (Figure 9D), p65 (Figure 9E) and CHOP (Figure 9F) reduced the expressions of cleaved caspase-3 and -9. These results agree with our observations in Figure 7, where we also showed reduction in cell death assessed via MTT assay and TUNEL staining. Together, these results suggested that increased expressions of cleaved caspase-3 and caspase-9 due to MA-mediated ER stress led to apoptotic cell death in astrocytes and this increase involved ATF6, IRE1α and PERK signaling pathway.

## DISCUSSION

Several studies have been undertaken to understand MA-associated neurotoxicity. MA primarily targets striatum and substantia nigra and disrupts dopaminergic system leading to various CNS symptoms. The neurotoxicity of MA is attributable to biochemical processes such as oxidative stress, excitotoxicity or pro-inflammatory cytokines/chemokines [50]. In addition to neurons, MA is also disturbs glial cell activity and thereby exhibit neurotoxic and addictive effects [51]. Several studies have demonstrated involvement of transcription factors, cytokine receptors, excitatory amino acid transporters (EAATs), glucose uptake mechanisms or oxidative stress in MA-mediated astrocyte toxicity [15, 16, 52, 53]. Despite these findings, the underlying cause for MA-mediated cell death in astrocytes is still unclear. We have shown in our recent study that MA increased oxidative stress and thereby led to caspase-3-mediated cell death in astrocytes [16]. In the current report, we further expanded our findings on MA-associated apoptotic mechanisms that involved ER stress. Our results demonstrated that MA-mediated activation of ATF6, IRE1α and PERK pathways differentially contributed to MA-mediated apoptosis.

As previously reported, MA is known to induce the expressions of several of ER-stress molecules in various regions of rat brains [28, 54]. Our findings are consistent with these prior reports in that we observed increase in various ER stress proteins in different brain regions of mice. In particular, we observed increase in all the key ER stress regulators in parietal cortex, which largely consist of astrocytes. This increase in ER stress due to MA in the parietal cortex could directly disrupt cognitive function and depressive like behavior [55, 56]. We also demonstrated this phenomenon in astrocyte cell-line and primary astrocytes, which is the first report showing cell specific MA-mediated ER stress in the brain. Our *in vitro* study demonstrated time-dependent increase in the RNA and protein levels of BiP, CHOP and XBP1 after single exposure. Physiologically, the accumulation of unfolded proteins in the ER triggers a protective response, wherein BiP chaperones the unfolded/misfolded proteins for ubiquitination [21, 22]. However, prolonged exposure to MA could produce sustained ER stress, which might impair the cells beyond repair. In this study, a single MA exposure triggered apoptosis (Figure 7), which suggests that the transient increase in BiP and CHOP observed in the present study was sufficient to generate a sustained ER stress. In an earlier study, low-dose MA has been shown to increase BiP but not CHOP in rat midbrains suggesting its protective effects via suppression of oxidative stress and apoptosis [54]. However, our study showed increase in both BiP and CHOP at various concentrations of MA. This difference could be partially explained by the fact that our *in vitro* study focused on astrocytes alone, while the earlier study utilized midbrain, which includes other cells in addition to astrocytes. Further, our study was performed over a period of time, while the previous study represents a cross-sectional study at a given time.

**Figure 10:**
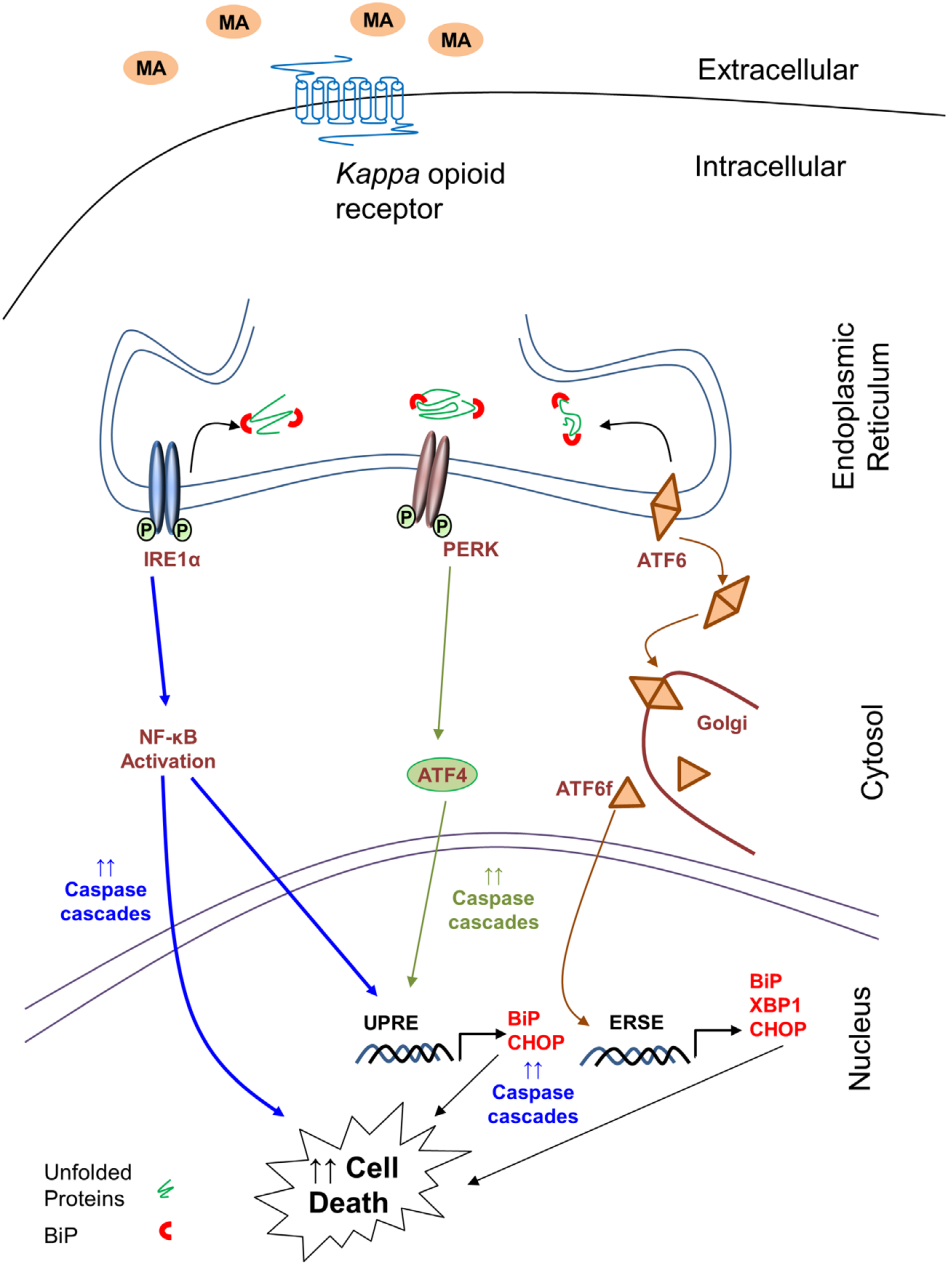
Schematic of signaling pathways involved in MA-mediated ER stress in astrocytes The signaling pathway sought in the present work is ER stress signaling cascade, which is responsible for MA-mediated cell death in astrocytes. The increase in the levels of BiP and CHOP suggest increased ER stress. This results in activation of ATF6, IRE1α and PERK pathways. Activation of ATF6 is signified by increased accumulation of ATF6 in golgi, where it is cleaved. The free cytosolic form of ATF6 then increases the transcription of BiP, CHOP and XBP1 via ER stress-response element (ERSE). Activation of IRE1α leads to activation of NF-κB, which further increases transcription of CHOP and engage various caspases to signal for apoptosis. Similarly, activation of PERK leads to increase in ATF4, which can increased transcription of BiP and CHOP via unfolded protein response element (UPRE). Finally, the convergent effect of these three mechanisms further accumulates CHOP leading to apoptosis via caspase cascades. Thus increase in ER stress due to MA activates the intrinsic caspase cascade via caspase-3 and caspase-9 cleavage. The kappa opioid receptor serves as an anchor for MA, which allows for the downstream activation of various events in the ER stress pathways. Finally, these cascades lead to cell death in astrocytes.

The increase in CHOP is a result of an orchestrated process, which involves activation of one or multiple of the three ER stress pathways (Figure 10). In the present study, we observed activation of all three pathways - ATF6, IRE1α and PERK. Previously, MA was shown to increase ATF6 mRNA via D1 receptor-dependent mechanism [26, 28]. Our study is in accordance with this report as we demonstrated increase in the protein levels of ATF6. In addition, we also demonstrated translocation of ATF6 and increase in cleaved ATF6 as a result of MA treatment (Figure 2). This confirmed the activation of ATF6 pathway since translocation of ATF6 from ER lumen to golgi signifies its activation. The splicing of ATF6 can only take place in the golgi, which is an important step for its transcriptional activity to increase ER chaperones and ER-associated protein degradation (ERAD) components [57–60]. We also observed reduction in the expressions of XBP1 and CHOP with ATF6 silencing, which further confirmed the activation of ATF6 pathway. It is known that ATF6 is responsible for transcriptional regulation of XBP1 [43] and our observation is along the similar lines, where in we demonstrated MA-mediated increase in XBP1 mRNA and protein, which were reduced with ATF6 silencing. Overall MA-mediated ATF6 activation is responsible for increased ER stress in astrocytes.

Apart from ATF6, IRE1α also increases the expressions of CHOP during ER stress. To our knowledge there is no report demonstrating MA-mediated activation of IRE1α pathway. In this study, we have demonstrated increased phosphorylation of IRE1α, which further activated NF-κB via phosphorylation of IκBα. In our earlier reports, we demonstrated involvement of NF-κB pathway in MA-mediated increase in pro-inflammatory cytokine/chemokine, IL-6 and IL-8 suggesting its involvement in neuroinflammation [15]. Our observation in the present study further consolidates the role of NF-κB pathway in MA-mediated cytotoxicity via induction of ER stress. It is known that XBP1 expression is increased via ATF6 but its splicing occurs via IRE1α [43]. It has also been shown in a previously published study that MA causes increase in XBP1 splicing in rat striatum [28]. However, we did not observe XBP1 splicing due to MA in SVGA or primary HFA cells (Supplementary Figure S2). It is possible that either neuron or microglia but not astrocyte might have contributed to XBP1 splicing in rat striatum. Thus, although the XBP1 mRNA is increased via ATF6 in our study, it is not spliced via IRE1α in response to MA treatment. To our surprise, XBP1 protein was slightly increased at 24 H (Figure 2D). Similar results were observed in HFA as shown in Figure 5D, where increase in spliced XBP1 was observed at 6 H. However, the protein lysates obtained from mouse brains did not show XBP1 splicing (Figure 6). The discrepancy between the levels of XBP1s observed at protein and RNA levels could be explained by possible role of post-translational modification of XBP1 protein [61]. As demonstrated by Yoshida et al., XBP1U forms a heterodimer with XBP1S during prolonged UPR and decreases its activity. This mechanism plays a significant role especially when UPR-mediated splicing of XBP1 mRNA is weakly activated [62]. Therefore, it is possible that slight increase in XBP1s protein could be due to post-translational modification of XBP1u. Apart from NF-κB activation and XBP1 splicing, IRE1α is also known to activate JNK and subsequently AP-1 [63–65]. In our present study, we did not observe activation of JNK (data not shown). Therefore, MA-mediated activation of IRE1α utilized activation of NF-κB but not JNK and AP-1, to increase CHOP.

As mentioned earlier, ER stress is also associated with activation of PERK pathway. Physiologically the activation of PERK leads to phosphorylation of Eukaryotic translation initiation factor 2A (eIF2α), which halts the global translation except ATF4 expression [22, 66–69]. In our study, we observed activation of PERK and ATF4, which further led to increased expressions of CHOP as evident by knockdown studies for ATF4 and PERK. This finding is in accordance with earlier study where increased ATF4 at mRNA and protein levels was observed in rat striatum after MA treatment [28]. Altogether, we demonstrated that MA-associated ER stress involved all three pathways leading to CHOP expression.

Opioid receptors have also been implicated in MA-associated neurotoxicity [46, 47]. KOR has been found to increase MA-mediated autophagy in Human umbilical vein cells (HUVECs) and thereby protect the cells from apoptosis [48]. On the other hand, inhibition of KOR in nucleus accumbence prevented MA self-administration via negative reinforcement mechanism [46]. Our results demonstrating suppression of CHOP and 3 major ER stress activators; ATF6, IRE1α and PERK with naltrexone and nor-BNI suggested that opioid receptors contributed in MA-mediated ER stress. These inhibitors also prevented MA-mediated cell death. Therefore, our study indicates that KOR may serve as a receptor for MA in astrocytes, and inhibition of KOR could prevent cytotoxic effects of MA in astrocytes.

In general, increased ER stress is predominantly a pro-survival mechanism [70], but prolonged stress conditions lead to apoptosis. Sustained increase of CHOP expression is known to induce cell death in a variety of cells [23, 29-31, 71]. The overexpression of CHOP leads to apoptosis [29, 30], whereas knockdown of CHOP has been shown to reduce ER stress-associated apoptosis [23, 31, 71]. Previously, we have also demonstrated that increase in CHOP correlated with apoptosis in astrocytes [65]. In spite of the fact that CHOP is induced via ATF6, IRE1α and PERK, apoptosis is largely attributed to IRE1α and PERK pathways [72]. Our results are in accordance with this notion, where we demonstrated MA-mediated cell death was reversed with siRNA against IRE1α, p65, CHOP, ATF4 and PERK. Although ATF6 also induces expressions of CHOP and XBP1 [73], the cytotoxic effects of ATF6 has not been clearly reported. In our study, we have observed suppression of MA-mediated CHOP with ATF6 siRNA, which also inhibited MA-mediated cell death (Figure 8). XBP1 is usually associated with cell-survival and overexpression of spliced XBP1 induces apoptosis [74]. Since we did not observe XBP1 spicing in MA-treated astrocytes, we believe that the pro-apoptotic nature of ATF6 in MA-mediated cell death could be largely attributed to the increase in CHOP.

Apoptosis via MA has been well documented in many studies in a variety of cells [75–78]. MA was previously shown to activate caspase-3, caspase-6, caspase-9 and caspase-12 resulting in neuronal apoptosis [13]. We have demonstrated in our earlier study that MA-mediated cell death involved caspase-3 activation in astrocytes [16]. In the present report, we observed increase in cleaved caspase-3 and caspase-9 but not caspase-8. Since caspase-8 is activated during extrinsic pathway (primarily associated with extracellular stimulus via ligand binding to the death receptors such as TNF receptor-1; TNFR1 and FAS-associated death domain; FADD) and caspase-3 and caspase-9 are activated during intrinsic pathway (primarily associated with mitochondrial dysregulations due to a variety of stress including ER stress) [49], we believe that MA-mediated apoptosis is largely attributable to the intrinsic caspase pathway. Use of various siRNA targeting ATF6, IRE1α and PERK pathways reduced the MA-mediated increase in cleaved caspase-3 and -9, which indicates that MA-mediated ER stress was indeed responsible for apoptotic cell death. We also observed reduction in MA-mediate apoptosis as a result of p65 silencing, which consolidates the role of NF-κB in MA-mediated toxicity. Since NF-κB activation leads to increase in pro-inflammatory cytokine/chemokines [15], cytotoxicity in brain microvascular endothelial cells via redox imbalance [79] and induce BBB dysfunction [80, 81], it is evident that it plays a multi-factorial role in MA-mediated cytotoxicity. The results presented in this study further supports the notion that MA employs several mechanisms that converge to NF-κB activation. Various pathways of ER stress further compound its toxic effects in astrocytes.

In summary, the results from our study provide additional insights in MA-associated toxicity in astrocytes. The detailed mechanism explained in the current study provides novel targets in the management of MA-mediated cytotoxicity. Especially, based on our results from opioid receptor antagonists, it is likely that naltrexone could be employed in MA-mediated ER stress. In future, the results from our study may also provide a new direction for development of therapeutic strategies to overcome MA-mediated neurotoxicity, which has a direct implication for MA abusers.

## MATERIALS AND METHODS

### Cells and reagents

All the experiments unless mentioned were performed using SVGA cells, which is an astrocyte cell line originally developed from human astrocytes [82]. The cells were grown in complete Dulbecco's modified Eagle's medium (DMEM) supplemented with 1% L-glutamine, 1% sodium bicarbonate, 1% non-essential amino acids, 10% heat-inactivated fetal bovine serum and 50 μg/ml gentamicin sulfate and cultured at 37°C in a humidified chamber containing 5% CO_2_. For experiments, cells were cultured in 12-well plates at 2.5 × 10^5^ cells/well in 1 ml medium to obtain a monolayer and the treatments were performed after overnight incubation. For experiments involving siRNA transfections, SVGA were plated at 6 × 10^5^ cells/well with 2 ml medium in 6-well plates. For treatment with MA, 500 μM was used as optimum concentration for all the experiments. Specific siRNA (ON-TARGETplus SMART pool) for BiP, CHOP, IRE1α, p65, PERK, ATF4, ATF6 and control siRNA were purchased from Dhramacon (Thermo Fisher Scientific Inc., Waltham, MA, USA). The antibody against p-PERK (Catalog # sc-32577) was purchased from Santa Cruz Biotechnology (Santa Cruz, CA, USA). Antibodies against glyceraldehyde 3-phosphate dehydrogenase (GAPDH) (Catalog # 2118s), phosphor IκBα (Caspase # 2078s), p65 (Catalog # 8242s), caspase-3 (Catalog # 9662s), cleaved caspase-3 (Catalog # 9664s), caspase-9 (Catalog # 9508s), cleaved caspase-9 (Catalog # 7237s), IRE1α (Catalog # 3294s), CHOP (Catalog # 2895s & 5554s), ATF4 (Catalog # 11815s) and BiP (catalog # 3177s) were purchased from Cell Signaling Technology (Danvers, MA, USA). The antibodies against phospho IRE1α (Catalog # ab124945), GFAP (catalog # ab53554), XBP1 (Catalog # ab37152) and ATF6 (Catalog # ab203119 & ab11909) were purchased from Abcam (Cambridge, MA, USA). Antibody against GM130 (golgi marker) (Catalog # 12480s) was obtained from Cell Signaling Technology (Danvers, MA, USA). The secondary antibodies against appropriate primary antibodies and the fluorophore conjugated secondary antibodies were purchased from Cell Signaling Technology (Danvers, MA, USA). Methamphetamine, and naltrexone (general opioid receptor) were obtained from Sigma (Sigma-Aldrich, St. Louis, MO, US), while nor-binaltorphimine hydrochloride (kappa opioid receptor) was obtained from R & D systems (R&D Systems, Inc. Minneapolis, MN, USA). Vectashield mounting medium with DAPI was obtained from Vector Laboratories (Vector Laboratories, Burlingame, CA). Specific primers for real-time RT-PCR were obtained from IDT (Integrated DNA Technologies, Coralville, IA, USA) and the 3-(4,5-dimethylthiazol-2-yl)-2,5-diphenyltetrazolium bromide (MTT) reagent was purchased from Fisher Scientific (Pittsburgh, PA, USA).

### Primary astrocytes

Human fetal astrocytes (HFA) were prepared as mentioned earlier [83] from the aborted fetal tissues obtained from Birth Defect Research Laboratory (BDRL), Seattle, WA. Briefly, the tissues were shipped on Hibernate^®^-E Medium (Catalog # A1247601, Thermo Fisher Scientific Inc., Waltham, MA, USA), which was processed in HBSS buffer until the astrocytes were dissociated into single cell suspension. The larger tissues were cleaned from blood contaminants while dissociating them into smaller pieces. Upon 3-4 rounds of cleaning/dissociation process, the tissues were passed through 25 ml pipettes followed by gradually smaller bore pipettes. Finally, the smaller clusters of cells were passed through 70 μm and 40 μm strainer to dissociate the clusters into single cell suspension. These cells were centrifuged at 300 X g for 10 minutes, the supernatants were removed and the cells were mixed with complete DMEM-medium with F12 supplement. These cells were then plated in flasks for 7-10 days without disturbing them followed by further passaging them into P1. HFA obtained from P1 were characterized by the expression of GFAP and >98% cells were found to be positive (data not shown). For experiments, HFA were used at 1 × 10^6^ cells for 12-well plates and the treatments were performed after 36 hours once monolayer of adhered astrocytes was formed.

### Animals and drug treatment

Male C57BL/6 at the age of 14-16 week were used to study the effect of acute MA treatment on ER stress proteins in various regions of the brain. These mice were obtained from Jackson Laboratories and bred in the UMKC-Laboratory Animal Research Core (LARC) facility. All the mice in the study were housed in a group of 3-5 animals per cage in a controlled environment with 12h light/dark cycle (lights off at 7:00 PM) and ad libitum access to food and water. This study was in accordance with the NIH guidelines and the experimental protocols were approved by the institutional animal care and use committee (IACUC) at UMKC. For the experiment, 4 age-matched mice each were grouped into saline and MA treatment. For acute treatment with MA, 4 s.c. injections of 10 mg/kg MA separated by 2 hours apart were given as per previous report [44]. The mice were caged for 24 hours after the last injection and the brains were collected after cardiac perfusion. The brain hemispheres were dissected into prefrontal cortex (PFC), parietal cortex (PC), hippocampus (Hippo), entorhinal cortex (EC) and cerebellum (Cer) for protein lysates and western blots were performed to estimate protein levels of various ER stress proteins.

### Transfection with siRNA

SVGA cells were transfected with siRNA as mentioned previously [16, 84]. Briefly, monolayer of 6 × 10^5^ cells in a 6-well plate was transfected with 20 nM of siRNA using Lipofectamine2000™ (Life Technologies, Carlsbad, CA, USA). The cells were washed twice with PBS to remove serum and supplemented with serum-free medium containing transfection cocktail. The transfected cells were incubated for 24 hours followed by replacement of the growth medium containing transfection cocktail with complete DMEM medium containing serum for additional 10 hours. The cells were then trypsinized, recounted and seeded at 2 X10^5^ cells per well in 12-well plates. The following day, cells were treated with or without MA to assess the silencing effect of specific target on MA-mediated ER stress. The efficiency of gene silencing was assessed using western blotting and found to be between 40-70% (Supplementary Figure S1).

### Real-time reverse transcriptase-polymerase chain reaction (RT-PCR)

The mRNA levels of BiP and CHOP expressions were determined using real-time RT-PCR. Briefly, SVGA cells were treated with MA for given time and upon termination of the treatment, total RNA was isolated using Qiagen RNeasy Mini Kit (Qiagen, Valencia, CA, USA). Next, 100 ng RNA was reverse transcribed at 37°C for 60 min followed by amplification of the target mRNA. The relative expressions of BiP was quantified using forward primer: 5′-CGAGGAGGAGGACAAGAAGG-3′ and reverse primer: 5′-AGTTCTTGCCGTTCAAGGTG-3′ with appropriate amplification conditions (Annealing at 62°C for 30 sec, denaturation at 95°C for 15 sec). Similarly, CHOP was measured using forward primer: 5′ GCACCTCCCAGAGCCCTCACTCTCC-3′ and reverse primer: 5′-CGCAGGGGGAAGGCTTGGAGTAGAC-3′ using pre-optimized amplification conditions (Annealing at 62°C for 30 sec, denaturation at 95°C for 15 sec). The levels of XBP1 were determined using primer sequences and amplification conditions as described previously [65]. All the expression values were normalized using Hypoxanthine-guanine phosphoribosyltransferase *(HPRT)* as a housekeeping gene. Relative fold expressions were analyzed using the 2^−ΔΔCt^ method.

### Western blotting

Detection of various signaling intermediates at protein levels was performed using western blotting in whole cell lysates. Briefly, upon termination of the treatments, growth medium was removed and cells were washed twice with phosphate buffer saline (PBS). The cells were then lysed in radioimmunoprecipitation assay (RIPA) buffer (Boston BioProducts, Ashland, MA, USA) supplemented with Halt™ protease inhibitor cocktail (Thermo Fisher Scientific Inc., Waltham, MA, USA) for 10 min at 4°C. The lysates were then homogenized for 30 sec and centrifuged at 14000 Xg for 10 min at 4°C to remove debris. The lysates were stored at −80°C until use.

The concentrations of protein lysates were measured using BCA protein assay kit (Thermo Fisher Scientific Inc., Waltham, MA, USA) and 20 μg protein was loaded on 10-12% SDS-polyacrylamide gel. The proteins were then resolved by electrophoresis at 80 V for 2.5 hours followed by transfer onto PVDF membranes at constant 350 mA current for 75 min at 4°C. Next, the membranes were blocked in 5% nonfat milk in PBST (0.075% Tween 20 in PBS) overnight at 4°C to reduce nonspecific signals. Specific target proteins were detected by incubating the membranes with appropriate concentrations of primary antibodies at room temperature for 2 – 2.5 hours. Upon incubation, the membranes were washed 5 times for 5 minutes each with PBST and again probed with respective horse radish peroxidase conjugated secondary antibodies for 2 hours at room temperature. Finally the membranes were washed again 5 times for 5 minutes each with PBST and target protein bands were visualized using BM chemiluminescence western blotting substrate (POD, Roche Applied Sciences; Indianapolis, IN, USA). The band intensities were quantified with FluorChem HD2 software (Alpha Innotech, San Leandro, CA, USA). Relative expressions of various proteins were calculated by normalizing the values of target proteins with GAPDH as loading control followed by comparison with control samples considered as baseline expressions. All western blots were performed with same experimental conditions and the loading controls were run for each blot individually. To precisely identify only target molecule, the membranes were cut between the molecular markers above and below the protein of interest.

### Immunofluorescent staining

The ATF6 translocation from ER to golgi was determined using immunocytochemistry. Briefly, 8 × 10^5^ SVGA cells were cultured on 1.5 mm coverslips in 6-well plates and treated with MA for 6 hours. After termination of treatment, the cells were fixed with ice-cold solution of 1:1 methanol-acetone for 20 min at −20°C. Next, the cells were air-dried and permeabilized with 0.3% Triton X-100 (PBST) for 10 min. Cells were rinsed thrice with PBS followed by blocking with 1% BSA in PBST for 30 min at room temperature. After blocking, the cells were incubated with a cocktail of rabbit anti-GM130 antibody (1:2500), mouse anti-ATF6 (1:500) and a goat anti-GFAP antibody (1:400) overnight at 4°C in humidified box. The cells were washed thrice with PBST followed by incubation with a cocktail of anti-mouse antibody conjugated with Alexa Fluor 555 (1:2000), anti-goat antibody conjugated with Alexa Fluor 647 (1:2000) and an anti-rabbit antibody conjugated with Alexa Fluor 488 (1:2000) (Cell Signaling Technology, Danvers, MA) in a dark chamber for 1 hour at room temperature. Finally, the cells were washed three times with PBS for 5 minutes each and the cover slips were mounted on a glass slide with 10 μl Vectashield mounting medium with DAPI. The images were captured using a 40X lens on Leica TCS SP5 II Laser Scanning Confocal microscope.

### Cell survival (MTT assay)

SVGA cells were seeded in 24-well plates at the density of 2.5 × 10^4^ cells/well and appropriate treatments were performed. After 48 hours of MA treatment, cell viability was determined using the colorimetric MTT reagent, which is converted into purple formazan crystals by mitochondrial dehydrogenase in the viable cells. Briefly, upon treatments, cells were treated with 0.2 mg/ml MTT solution prepared in serum free medium and incubated at 37°C for 3-4 hours. The labeling was terminated by carefully removing MTT reagent followed by cell lysis with 300 μl dimethylsulfoxide, which dissolves the formazan crystals to form a clear purple solution. The color intensity was measured using Benchmark Microplate Reader (Bio-Rad Laboratories, Hercules, CA, USA) with absorbance at 570 nm with a reference filter at 650 nm. Cell viability was calculated with the absorbance in control cells as 100%.

### Terminal deoxynucleotidyl transferase dUTP nick end labeling (TUNEL) based apoptosis assay

The measurement of cell viability was correlated with DNA damage using TUNEL based apoptosis assay using TACS-XL *In Situ* Apoptosis Detection Kit - DAB kit as per manufacturer's protocol (Trevigen Inc., Gaithersburg, MD, USA). Briefly, 2 × 10^5^ cells/well were seeded in 12-well plates and the cells were exposed to the treatments with appropriate compounds. For experiments involving siRNA, the transfections were performed as mentioned earlier followed by treatment with MA for 48 hours. After treatment termination, cells were washed with PBS, air-dried for 5 min and fixed with 3.7% formalin in PBS for 10 min at room temperature. After fixation, they were incubated for 10 min at RT with Cytonin™ solution to permeabilize the cells. Next, cells were blocked in 3% H_2_O_2_ in methanol for 5 min, washed and labeled with 1x TdT labelling mixture (dNTP mix + TdT enzyme + cationic buffer + TdT labelling buffer) for 1 hour. The labelled cells were washed twice and incubated with Strep-HRP antibody solution for 30 min at 37°C. Next, cells were incubated for a reaction with DAB solution for 15 min. The smear of cells was washed with PBS to remove excess of labeling reagents and then observed under light microscope. The images were taken using Labomed iVu5100 (Labo America Inc., Fremont, CA, USA).

### Statistical analysis

The statistical analysis was performed and all the data were reported in mean ± S.E. values. All the results were confirmed in at least three separate experiments unless specified, with each experiment performed in triplicates. For the comparison between mock/control group and different treatments, two-tailed Student's t-test was applied to calculate P-values, and P-value ≤0.05 (*) or ≤0.01 (**) was considered statistically significant. Experiments containing multiple variables such as pharmacological inhibitor or siRNA were analyzed using one-way ANOVA with multiple comparisons to address whether the suppression was significant. The western blots shown in each panel demonstrate a representative blot, where each experiment was performed at least 3 times and mean ± S.E. of band intensities were plotted in the bar diagrams. No adjustments were made in the statistical analysis for multiple comparisons.

## SUPPLEMENTARY FIGURES


