# Supplementary material for: Methamphetamine-mediated endoplasmic reticulum (ER) stress induces type-1 programmed cell death in astrocytes via ATF6, IRE1α and PERK pathways
Source: Oncotarget. 2016 Jun 14;7(29):46100–19. doi: 10.18632/oncotarget.10025 (PMC5216784; doi:10.18632/oncotarget.10025)
Supplement: Supplementary file 1 [file oncotarget-07-46100-s001.pdf]

Methamphetamine-mediated endoplasmic reticulum (ER) stress induces type-1 programmed cell death in astrocytes via ATF6, IRE1α and PERK pathways

SUPPLEMENTARY FIGURES

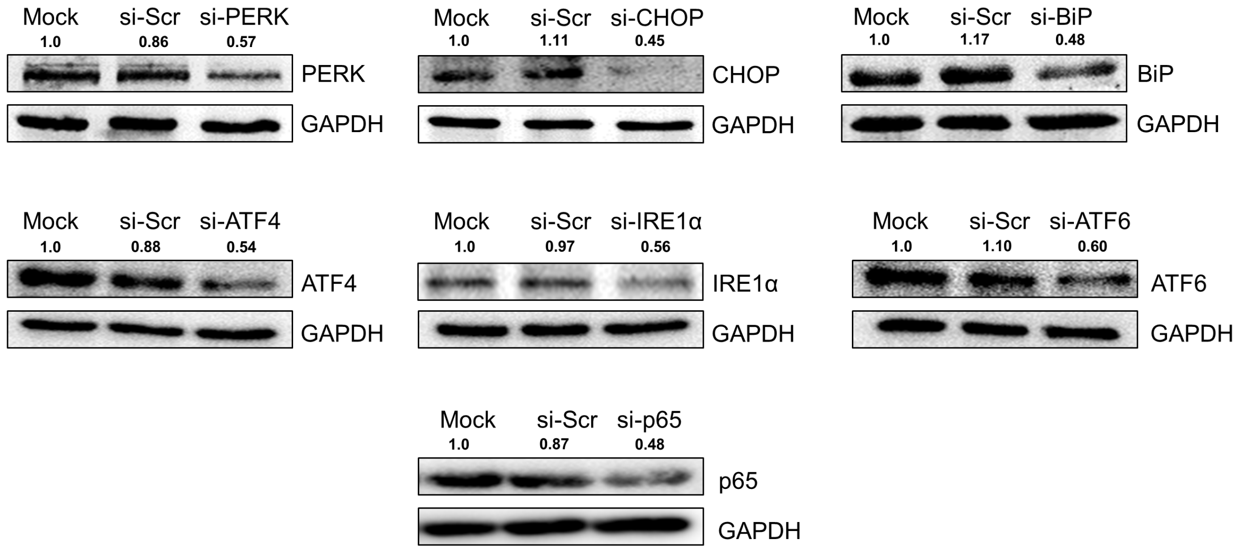

Supplementary Figure S1: The efficiency of siRNA-mediated silencing of the target molecules. SVGA cells were transfected with 20 nM of siRNA in a 6-well plate as described in Methods section. After 48 hours, the cells were lysed with lysis buffer and protein expressions were determined with western blotting. The western blots are representative images and the numbers above the blots represent mean intensity of the respective bands. The blots presented in the figures were obtained by cutting membranes at the molecular markers above and below protein of interest before probing them for appropriate primary and secondary antibodies. The images are then presented as is with brightness/contrast adjustment applied throughout the blot without altering the overall results.

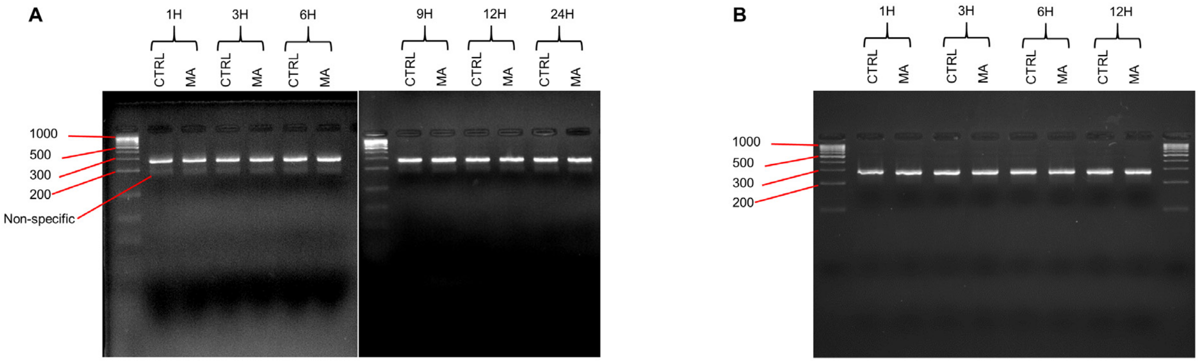

Supplementary Figure S2: MA treatment does not induce splicing of XBP1 mRNA in astrocyte cells and HFA. A. SVGA cells were seeded at 2.5 × 10^5 cells/well in 12-well plates and treated with 500 µM MA for 1, 3, 6, 9, 12 & 24 H. Upon treatment, the cells were harvested and RNA were isolated using RNeasy mini kit. XBP1 RNA were amplified for both, unspliced (289 bp) and spliced (263 bp) fragments and the product was resolved using 3.5% agarose gel as described in [65]. **B.** HFA cells were seeded at 1 X 10^6 cells/well in 12-well plates and treated with 500 µM MA for 1, 3, 6 & 12 H. Similar to SVGA astrocytes, the RNA were isolated and were amplified for XBP1. The amplified product was resolved using 3.5% agarose gel. The blots shown are representative images of at least 3 independent experiments for SVGA and 3 donors for HFA.
